# Exploring factors associated with non-alcoholic fatty liver disease using longitudinal MRI

**DOI:** 10.1186/s12876-024-03300-0

**Published:** 2024-07-23

**Authors:** Friedrich Horn, Till Ittermann, Marie-Luise Kromrey, Danilo Seppelt, Henry Völzke, Jens-Peter Kühn, Felix Schön

**Affiliations:** 1 Institute of Diagnostic Radiology and Neuroradiology, University Medicine Greifswald, Greifswald, Germany; 2 Institute for Community Medicine, University Medicine Greifswald, Greifswald, Germany; 3 Institute and Policlinic for Diagnostic and Interventional Radiology, Faculty of Medicine and University Hospital Carl Gustav Carus, Technische Universität Dresden, Dresden, Germany

**Keywords:** Fatty liver, Metabolic syndrome, Longitudinal study, Magnetic resonance imaging

## Abstract

**Background:**

To identify factors associated with non-alcoholic fatty liver disease over a 5-year period.

**Methods:**

Three hundred seven participants, including 165 women, with a mean age of 55.6 ± 12.0 years underwent continuous quantitative MRI of the liver using the proton-density fat fraction (PDFF). The liver’s fat fractions were determined at baseline and 5 years later, and the frequency of participants who developed fatty liver disease and potential influencing factors were explored. Based on significant factors, a model was generated to predict the development of fatty liver disease.

**Results:**

After excluding participants with pre-existing fatty liver, the baseline PDFF of 3.1 ± 0.9% (*n* = 190) significantly increased to 7.67 ± 3.39% within 5 years (*p* < 0.001). At baseline, age (OR = 1.04, *p* = 0.006, CI = 1.01–1.07), BMI (OR = 1.11, *p* = 0.041, CI = 1.01–1.23), and waist circumference (OR = 1.05, *p* = 0.020, CI = 1.01–1.09) were identified as risk factors. Physical activity was negatively associated (OR = 0.43, *p* = 0.049, CI = 0.18–0.99). In the prediction model, age, physical activity, diabetes mellitus, diastolic blood pressure, and HDL-cholesterol remained as independent variables. Combining these risk factors to predict the development of fatty liver disease revealed an AUC of 0.7434.

**Conclusions:**

Within a five-year follow-up, one-quarter of participants developed fatty liver disease influenced by the triggering factors of age, diabetes mellitus, low HDL-cholesterol, and diastolic blood pressure. Increased physical activity has a protective effect on the development of fatty liver.

**Supplementary Information:**

The online version contains supplementary material available at 10.1186/s12876-024-03300-0.

## Introduction

Non-alcoholic fatty liver disease (NAFLD) is becoming increasingly prevalent with the rise of obesity and metabolic syndrome [1]. Some authors even consider NAFLD to be a hepatic manifestation of metabolic syndrome [2]. A study conducted in the United States revealed that 30.0% of subjects had NAFLD [3], while an even higher prevalence of 42.2% was reported in Northeastern Germany in the same year [4]. The presence of NAFLD has been shown to increase medical treatment utilization and healthcare costs by 26% per year compared to the general population [5].

NAFLD increases all-cause mortality, primarily due to liver-related and cardiovascular causes. Additionally, NAFLD doubles the incidence of type 2 diabetes mellitus (T2DM) [6] and chronic kidney disease [7]. NAFLD is also considered an independent risk factor for the development of colorectal adenoma [8], a precursor lesion of colorectal cancer in the adenoma-carcinoma sequence [9]. Furthermore, NAFLD appears to be associated with an increased risk of ischemic stroke [10].

Simple NAFLD, in which there is no damage to the hepatocytes, can progress to the more aggressive non-alcoholic steatohepatitis (NASH) in approximately 20% of cases [11]. In NASH, there is inflammation characterized by damage to the hepatocytes leading to fibrosis [12]. Liver-related diseases can develop in the course of NASH, including cirrhosis, portal hypertension, or even liver cancer. NASH often goes undiagnosed for a long time and only manifests itself when symptoms such as ascites, sudden oesophageal variceal bleeding or the development of hepatocellular carcinoma occur [13].

Due to the increasing prevalence of obesity, metabolic syndrome and NAFLD, it is important to detect NAFLD at an early stage or to identify the triggering factors in order to take appropriate preventive measures. To our knowledge, there are no studies that have examined potential risk factors for the development of fatty liver through a longitudinal survey. Therefore, the aim of this study is to identify factors associated with NAFLD and to develop a risk score predicting the development of NAFLD over a 5-year period.

## Materials and methods

### Study population

**Fig. 1:**
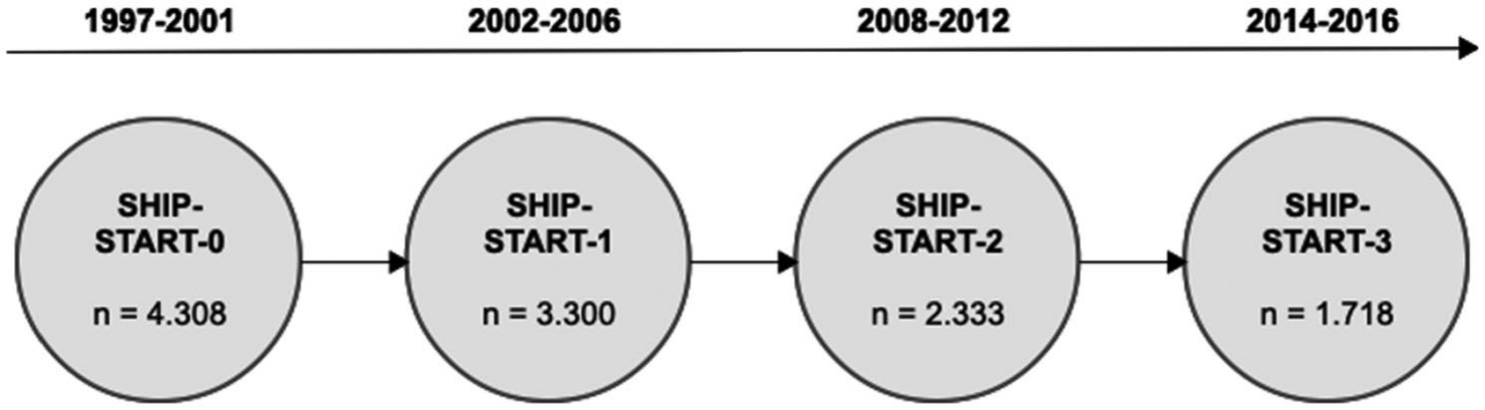
Phases of the SHIP Study in Northeast Germany between 1997 and 2016, consisting of a baseline and three follow-up courses

Participants in this study were recruited from the Study of Health in Pomerania (SHIP), a population-based study in Northeastern Germany. A random sample of 213,057 Caucasian inhabitants was drawn for recruitment. From 1997 to 2001, the first cohort, SHIP-START-0, consisted of 4,308 adult subjects out of a net sample of 6,265 individuals aged 20–79 years. The first follow-up survey, SHIP-START-1, was conducted between 2002 and 2006, and the second follow-up, SHIP-START-2, took place between 2008 and 2012. Whole-body magnetic resonance imaging (MRI) was performed for the first time in SHIP-START-2, and between 2014 and 2016, a further follow-up of participants included in SHIP-START-2 was conducted, known as SHIP-START-3, which also involved the use of whole-body MRI [4, 14–16]. An overview of the SHIP study, including follow-ups, is shown in Fig. 1. The ethics committee of the University of Greifswald approved all experiments, including the Study of Health in Pomerania and their follow-ups, and informed consent was obtained from all subjects. The datasets used and/or analyzed during the current study are available from the corresponding author upon reasonable request (https://www.fvcm.med.uni-greifswald.de).

**Fig. 2:**
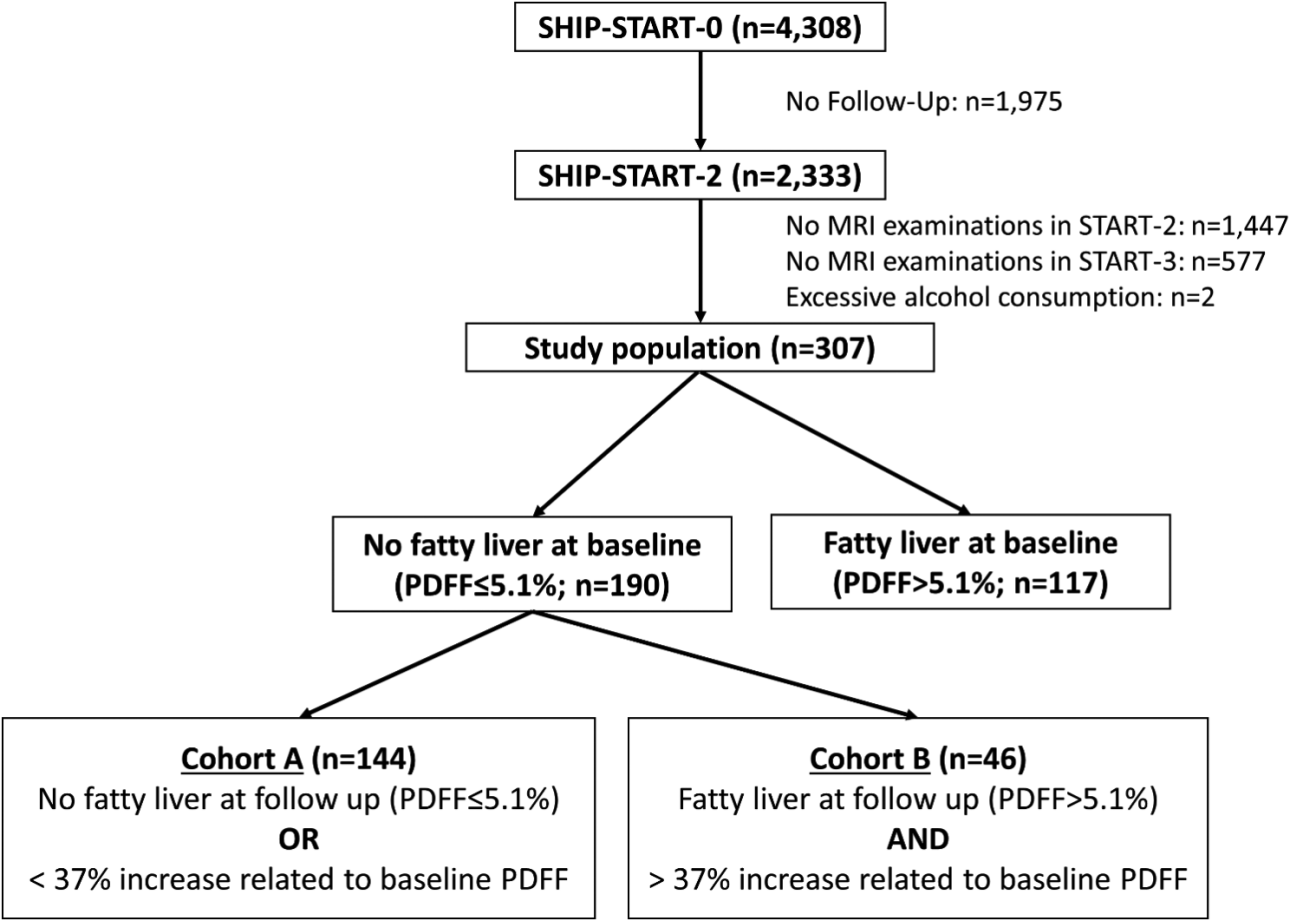
The flow-chart figures the recruitment of the whole study population from the SHIP-cohort. For further statistical analyses (Cohort A versus Cohort B), only participants without fatty liver disease at baseline were included

For this project, participants from the SHIP-START-2 and SHIP-START-3 cohorts who underwent a quantitative MRI of the liver were included. The SHIP-START-2 cohort is designated as the baseline, and the SHIP-START-3 cohort is referred to as the 5-year follow-up. Out of the 2,333 individuals examined in SHIP-START-2, data on liver fat content were available for 886 participants, of whom 577 had no measurement on liver fat content available at follow-up and were therefore excluded (see Fig. 2). After excluding two individuals who reported consuming more than 60 g/day of alcohol, the final study population consisted of 307 participants, including 165 women and 142 men, with a mean age of 55.6 ± 12.0 years (range 31–82 years).

### Imaging and assessment of fatty liver disease

MRI scans were performed at baseline and follow-up on the same 1.5 Tesla MRI scanner (Magnetom Avanto, Siemens Healthcare AG, Erlangen, Germany), using a 12-channel phased-array coil. Both cohorts underwent 3D multi-echo-chemical shift encoded gradient echo sequences to cover the upper abdominal organs. Sequence details are presented in Table 1.

Liver fat content was assessed using the proton-density fat fraction (PDFF) technique, which was calculated by post-processing the MRI data using a homemade Matlab software algorithm (version 2011a, Mathworks, Natick, MA, USA). Our fat fraction was corrected for T1 bias, T2* decay and for the multispectral complexity of fat [17, 18]. PDFF is an accurate and reliable technique to quantify liver fat, with no software or hardware variability. The details of the MRI data reconstruction are described elsewhere [19].

For the quantitative measurement of liver fat, the observers drew a region of interest (ROI) around the liver parenchyma in a representative slice of the PDFF map, omitting large vessels, artifacts, or lesions if possible. Image analysis was performed using Horos software (Horosproject.org, v3.3.6, Nimble Co LLC d/b/a Purview in Annapolis, MD, USA). Observer 1 (JPK), a radiologist with 14 years of experience in reporting MRI, reviewed all datasets from SHIP-START-2, while observer 2 (FH), a trained medical student, analyzed the SHIP-START-3 data.

### Group definition

At baseline, the study population was divided into two groups based on the previously published threshold value of PDFF 5.1%, which defines the presence or absence of fatty liver disease [19]. Moreover, the severity of hepatic steatosis was classified into mild (> 5.1–14.0%), moderate (> 14.0-28.0%), and severe (> 28.0%) based on previously established cut-off values [19].

From the initial study population, 117 participants had fatty liver disease and were excluded from further analysis, leaving 190 subjects without fatty liver disease. From this group, two additional cohorts were formed based on the development of fatty liver disease and the degree of increase in liver fat content during follow-up. Fatty liver disease development was defined as a PDFF > 5.1%, and only individuals with a 37% increase in liver fat content from baseline were considered to have a substantial increase in liver fat. The 37% increase represents the median change in PDFF between baseline and follow-up. Individuals meeting these criteria were assigned to Cohort B, “relevant fatty liver”, while all others were assigned to Cohort A, “no fatty liver”, to increase group differentiation (see Fig. 2). Moreover, this definition helps to mitigate the bias associated with participants who had initially borderline PDFF values.

However, the Quantitative Imaging Biomarkers Alliance (QIBA) considers a change in PDFF of ± 5% to be relevant [20]. A corresponding calculation with a cut-off value of 5% (instead of 37%) can therefore be found in the supplements (Appendix).

### Clinical data

Demographic and clinical data were collected exclusively at baseline to identify potential risk factors associated with the development of fatty liver disease. Demographic variables such as age, sex, and body mass index (BMI) were recorded, while clinical and behavioral factors such as waist circumference, systolic and diastolic blood pressure, alcohol consumption, smoking status, physical activity level, presence of type 2 diabetes, and dietary patterns were investigated as potential triggers for liver fat accumulation. Detailed descriptions of the definitions of these clinical and behavioral factors are provided in the supplementary documents (Appendix). In addition, laboratory data including glucose, cholesterol, low-density lipoprotein-cholesterol (LDL-C), high-density lipoprotein-cholesterol (HDL-C), triglycerides, alanine aminotransferase (ALT), aspartate aminotransferase (AST), and gamma-glutamyltransferase (GGT) were also assessed.

### Statistics

Liver fat content at baseline and follow-up was reported as percentage +/- standard deviation. Clinical characteristics of the study population collected at baseline were presented as mean and standard deviation for continuous data or as absolute numbers and percentages for categorical data, stratified by the subgroups (Cohort A/B). Logistic regression models, adjusted for age, sex, and baseline liver fat content, were used to investigate associations of baseline metabolic biomarkers with incident fatty liver (Cohort B vs. Cohort A).

Additionally, a logistic regression model for incident fatty liver (Cohort B vs. Cohort A) was constructed with all demographic, clinical, and laboratory variables as independent variables that had a p-value < 0.2 in the single analysis. A backward elimination procedure was applied so that only variables with a p-value < 0.1 were included in the final model. The area under the curve (AUC) of the receiver operating characteristic (ROC) analysis was used to assess the model’s discrimination.

A p-value < 0.05 was considered statistically significant. All analyses were performed with Stata 16.1 (Stata Corporation, College Station, TX, USA).

## Results

At baseline, the mean PDFF of the entire study population was 6.80 ± 6.52%. Among the 117 subjects with fatty liver disease at baseline, 80 had mild, 31 had moderate, and 6 had severe fatty liver disease. One hundred and ninety participants had no fatty liver at baseline, with a mean fat content of 3.1 ± 0.9%. The healthy population at baseline consisted of 77 men and 113 women with a mean age of 55.6 +/- 12.0 years. During follow-up, the mean PDFF increased to 8.18% in the entire study population (*p* < 0.001) **(**Table 2**)**. Of the 190 individuals with a baseline PDFF ≤ 5.1%, 144 individuals did not develop fatty liver disease during follow-up (Cohort A; mean PDFF 3.49 ± 1.06). In contrast, 46 participants (24.2%) developed significant fatty liver disease (Cohort B; 7.67 ± 3.39). Of these, 43 subjects developed mild and three developed moderate steatosis hepatis according to the above-mentioned cut-off values. Nonetheless, in both cohorts A and B, liver fat content increased significantly during follow-up.

**Table 1:** MRI sequence details at Baseline and Follow-Up examination

	Baseline	Follow-Up
TR/TE in ms	TR: 11 / TE: 2.4, 4.8, 9.6	TR: 12 / TE: 2.4, 4.4, 6.4, 8.4, 10.5
Flip Angle in °	10	5
Bandwidth in Hz/pixel	1065	1953
matrix	224 × 126 × 32	256 × 128 × 80
slice thickness in mm	6	5
parallel imaging acceleration factor	yes, 1.5	yes, 1.5
sequence length	19 s	19 s
orientation	transversal	coronar

**Table 2:** Liver fat content (PDFF in %) of baseline and follow-up in percent +/- standard deviation, n = number of subjects. Cohort A: no fatty liver in follow-up, Cohort B: relevant fatty liver in follow-up

	Whole population	Participants without fatty liver	Cohort A	Cohort B
	Baseline	Baseline	Follow-Up	
Numbers	307	190	144	46
Baseline (PDFF in %)	6.80 ± 6.52	3.10 ± 0.90	2.97 ± 0.92	3.48 ± 0.76
Follow-up (PDFF in %)	8.18 ± 6.94	4.50 ± 2.61	3.49 ± 1.06	7.67 ± 3.39
p-value	*p* < 0.001	*p* < 0.001	*p* < 0.001	*p* < 0.001

Subjects in Cohort B who developed fatty liver within the 5-year study period were, on average, older and more obese than participants in Cohort A who did not develop fatty liver **(**Table 3**)**. Significant differences between the groups were found in age (OR = 1.04, *p* = 0.006, CI = 1.01–1.07), BMI (OR = 1.11, *p* = 0.041, CI = 1.01–1.07), and waist circumference (OR = 1.05, *p* = 0.020, CI = 1.01–1.09). Conversely, physically active individuals (OR = 0.43, *p* = 0.049, CI = 0.18–0.99) had a significantly lower likelihood of developing fatty liver compared to physically inactive individuals.

**Table 3:** Characteristics of participants without fatty liver disease collected at baseline and corresponding associations for participants who did not develop fatty liver (Cohort A) and developed fatty liver in follow-up (Cohort B). Each variable was tested independently. OR = odds ratio, p = level of significance

	Cohort A: No fatty liver in follow-up, *n* = 144	Cohort B: Relevant fatty liver in follow-up, *n* = 46	OR	*p*	95% Confidence interval
Age (years)	**52.30 ± 12.24**	**58.65 ± 11.98**	**1.04**	**0.006**	**1.01–1.07**
BMI (kg/m^2)	**25.90 ± 3.58**	**27.49 ± 3.62**	**1.11**	**0.041**	**1.01–1.23**
Waist circumference (cm)	**83.81 ± 10.42**	**90.45 ± 9.54**	**1.05**	**0.020**	**1.01–1.09**
Food frequency score	14.20 ± 3.23	14.78 ± 3.08	1.06	0.337	0.94–1.20
Alcohol consumption (g/day)	9.19 ± 10.83	7.64 ± 10.28	0.96	0.073	0.93-1.00
Glucose i.S. (mmol/l)	5.35 ± 0.68	5.33 ± 0.83	0.85	0.532	0.51–1.41
Cholesterol i.S. (mmol/l)	5.43 ± 1.11	5.59 ± 1.04	1.07	0.687	0.77–1.49
LDL-Cholesterol i.S. (mmol/l)	3.29 ± 0.82	3.48 ± 0.87	1.17	0.466	0.77–1.79
HDL-Cholesterol i.S. (mmol/l)	1.59 ± 0.37	1.46 ± 0.32	0.35	0.063	0.11–1.06
Triglycerides (mmol/l)	1.36 ± 0.74	1.75 ± 0.96	1.39	0.131	0.91–2.13
ALT i.S. (µmol/sl)	0.37 ± 0.20	0.39 ± 0.12	1.22	0.849	0.15–9.81
AST i.S. (µmol/sl)	0.29 ± 0.11	0.30 ± 0.11	0.71	0.842	0.03–19.78
GGT (µmol/sl)	0.55 ± 0.43	0.67 ± 0.75	1.26	0.452	0.69–2.31
Systolic blood pressure (mm/Hg)	126 ± 17	134 ± 16	1.01	0.273	0.99–1.04
Diastolic blood pressure (mm/Hg)	78 ± 9	82 ± 9	1.04	0.089	0.99–1.08
Smoking status					
former	51 (35.7%)	20 (43.5%)	0.97	0.934	0.42–2.22
current	32 (22.4%)	8 (17.4%)	1.14	0.790	0.41–3.18
Arterial hypertension	53 (36.8%)	27 (58.7%)	1.49	0.306	0.70–3.18
Male gender	53 (36.8%)	24 (52.2%)	1.60	0.184	0.80–3.19
Diabetes known yes	1 (0.7%)	4 (8.7%)	8.99	0.064	0.88–92.03
Physical activity yes	**115 (80.4%)**	**32 (69.6%)**	**0.43**	**0.049**	**0.18–0.99**

Data are expressed as mean ± standard deviation (continuous variables) or as absolute numbers and percentages (categorical variables). Odds ratios (OR) are derived from logistic regression models adjusted for age, sex, and liver fat content at baseline.

In our prediction model for incident fatty liver, the independent variables that remained in the final model were age, physical activity, known diabetes mellitus, diastolic blood pressure, and HDL-cholesterol **(**Table 4**)**.

**Table 4:** Variables kept in the final prediction model for incident fatty liver

	Odds Ratio	*p*	95% Confidence interval
Age	**1.06**	**0.001**	**1.02–1.09**
Physical activity yes	**0.34**	**0.015**	**0.14–0.82**
Diabetes mellitus yes	**10.10**	**0.074**	**0.80–127.4**
Diastolic blood pressure	**1.05**	**0.011**	**1.01–1.10**
HDL-cholesterol i.S.	**0.28**	**0.027**	**0.09–0.87**

Results are derived from a logistic regression model.

The formula for calculating the individual risk for incident fatty liver is:

“1/(1 + 1/exp(-5.77913 + 0.05516*age (years) – 1.06808 (if physically active) + 2.31271 (if having type 2 diabetes) + 0.05353*diastolic blood pressure (mmHg) – 1.27004*HDL-cholesterol))”.

*An example for a hypothetical 20-year-old person who is physically active and has no type 2 diabetes mellitus is as follows*:

*The formula to calculate the individual risk of developing a fatty liver within 5 years is*:

1 / (1 + exp(-5.77913 + 0.05516 * age + (-1.06808) * (physical activity = yes) + 0 * (diagnosed type 2 diabetes mellitus = no) + 0.05353 * diastolic blood pressure + (-1.27004) * HDL-cholesterol)).

*Plugging in the values, we get:*

1 / (1 + exp(-5.77913 + 0.05516 * 20–1.06808 + 0 + 0.05353 * 80–1.27004 * 1.6)) = 0.029.

*Therefore, the individual risk of developing a fatty liver within 5 years is 2.9%.*

*An example for a hypothetical 79-year-old person who is physically inactive and diagnosed with type 2 diabetes mellitus is as follows*:

1 / (1 + exp(-5.77913 + 0.05516 * age − 0 * (physical activity = no) + 2.31271 * (diagnosed type 2 diabetes mellitus = yes) + 0.05353 * diastolic blood pressure − 1.27004 * 1.22 (HDL-cholesterol in mmol/l))).

*Plugging in the values, we get:*

1 / (1 + exp(-5.77913 + 0.05516 * 79 − 0 + 2.31271 + 0.05353 * 90–1.27004 * 1.22)) = 0.985.

*Therefore, the individual risk of developing a fatty liver within 5 years is 98.5%.*

**Fig. 3:**
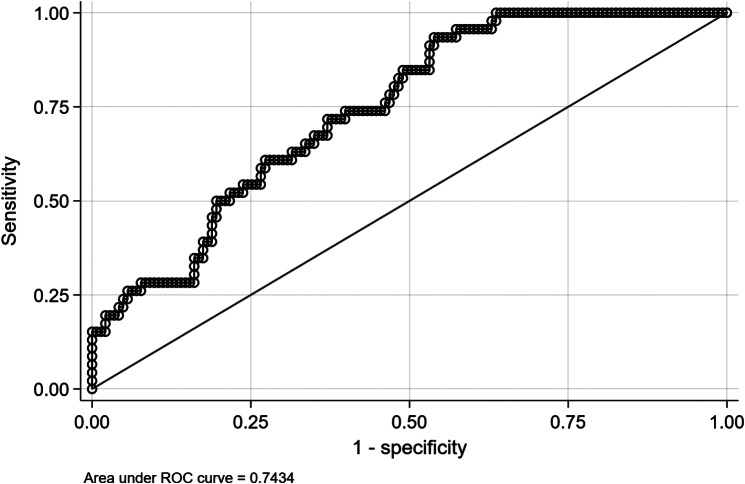
Area Under the Curve (AUC) of the final prediction model for incident fatty liver, defined as the combined presence of a PDFF > 5.1% and an increase in liver fat by 37% from baseline. The reported AUC value is 0.7434 (95% Confidence interval: 0.6696–0.8172)

The discrimination of the model was evaluated using ROC analysis, and the AUC was found to be 0.7434 (CI = 0.6696–0.8172) **(**Fig. 3**)**.

As stated in the methodology section, additional results - defined as individuals with a PDFF > 5.1% and an increase of 5% in liver fat content from baseline - can be found in the supplements (Appendix).

## Discussion

Our study aimed to investigate factors associated with the development of non-alcoholic fatty liver disease in a long-term study. We found that approximately 25% of healthy volunteers developed fatty liver disease within a 5-year period. Factors such as age, waist circumference, BMI, the presence of diabetes mellitus, and diastolic blood pressure were identified as triggering factors. Conversely, physical activity and HDL-cholesterol were negatively associated with the development of fatty liver.

The prevalence of fatty liver disease varies widely between studies, ranging from 20 to 30% [21–26] globally up to 42% [4] in northeastern Europe. However, the method used to detect fatty liver plays a critical role in determining its prevalence. Quantitative MRI has proven to be a reliable method for detecting fatty liver [27, 28]. In population-based cohorts using MRI, the prevalence of fatty liver ranged from 19 to 34% [4, 29–31] and up to 61% in exclusively obese subjects [29]. At baseline, we detected a frequency of 38.2% in our study population, which is consistent with published frequencies of fatty liver detected by MRI in the normal Caucasian population.

Additionally, we found an increase in liver fat content over a 5-year interval. To our knowledge, there are currently no published studies investigating the incidence of fatty liver over such a long-term period. Our study revealed that a quarter of the initially healthy population developed fatty liver within this 5-year interval.

There are numerous studies emphasizing the clinical significance of NAFLD and its associated complications, such as T2DM [6], chronic kidney disease [7], ischemic stroke [10], liver cirrhosis and hepatocellular carcinoma [13], among others. However, the etiology of NAFLD and the factors or combination of factors that contribute to its development remain poorly understood. Consistent with our findings, Alqahtani and Schattenberg et al. reported an association between increasing age and NAFLD prevalence [21]. Likewise, Stefan et al. suggested that aging could play an important role in the development of fatty liver [32].

Our investigation also highlighted increased waist circumference as an influencing factor. Similarly, Alqahtani and Schattenberg et al. reported an increased prevalence of NAFLD in patients with elevated central obesity, which is also related to NAFLD severity [21]. Cotter and Rinella et al. stated that truncal obesity is a more important determinant of NAFLD risk than BMI [33], which we confirmed in our longitudinal study.

Additionally, we found T2DM to be a strong triggering factor. Targher et al. described also T2DM as risk factor for faster progression of NAFLD to non-alcoholic steatohepatitis and identified NAFLD as a risk factor for incident T2DM [34]. Younossi et al. reported in their meta-analysis that T2DM is an important risk factor for NAFLD and seems to accelerate the progression of liver disease in NAFLD [35]. They also estimated the prevalence of metabolic co-morbidities among T2DM patients with NAFLD, with almost 60% having hypertension. In support, Jarvis et al. reported in their meta-analysis that in addition to lipid abnormalities, hypertension is independently associated with incident severe liver disease [36]. We established a correlation for high diastolic blood pressure in our investigation and found an inverse association for HDL-C with the development of NAFLD. Peng et al. reported that reduced HDL-C was significantly more common in the mild and moderate-to-severe NAFLD groups than in the control group without fatty liver [37]. Nass et al. showed the same result, with HDL-C being lower in subjects with non-alcoholic hepatic steatosis than in those without NAFLD [38].

We demonstrated that reduced physical activity is directly correlated with the development of liver fat or, conversely, that physical activity prevents the development of fatty liver. In this aspect, Babu et al. concluded that exercise overall likely had a beneficial effect on alleviating NAFLD without significant weight loss [22]. Gerage et al. suggested that even relatively moderate physical activity of ≥ 150 min/week for about 31 months has beneficial effects to influence the severity of hepatic steatosis [39].

Using the results of logistic regression, which include the following parameters: age, physical activity, known diabetes mellitus, diastolic blood pressure, and HDL cholesterol, we can predict the probability of fatty liver occurrence within the next 5 years. This prediction of fatty liver represents the novelty of this manuscript. With the formula presented here, it is possible to predict the development of fatty liver in the next years. Targeted prevention, including increased physical activity, can help prevent the development and progression of fatty liver.

The strengths of our study are the population-based approach and the long observation period of five years, allowing us to identify factors associated with the development of fatty liver and to predict the development of fatty liver disease over the next few years.

The limitations of our study include the relatively small number of subjects. This is mainly due to the fact that we selected subjects who developed a relevant fatty liver during the study period in order to compare them with subjects who maintained a stable low liver fat content during the observation period. In addition, Cohort A, consisting of healthy subjects, showed a significant increase in PDFF over the observation period, and it is likely that some of these subjects will also develop fatty liver over time. We believe that mild fatty degeneration may correspond to the physiological aging process. As a result, some parameters that have been suggested in the literature as influencing factors, such as high ALT, did not show statistically significant differences between the groups compared [4].

To ensure that the development of fatty liver in our study was not influenced by alcohol, we excluded subjects with an alcohol consumption of more than 60 g/day. This is supported by the non-significant association between alcohol consumption at baseline and the development of liver fat during follow-up. However, our study population consisted of white Caucasian subjects, so the findings may not be applicable to the general population.

Another limitation is that MRI measurements between baseline and follow-up were obtained using slightly different scan parameters. To address this issue, we used PDFF as a standardized MRI-based biomarker of tissue fat concentration that is reproducible across different MRI systems and scan parameters [40]. However, it is important to account for known confounding variables such as T1, T2* bias, noise error, and multispectral complexity of fat to accurately determine PDFF. Both the baseline PDFF and the follow-up PDFF calculations were carried out considering these limiting factors [15, 17, 41, 42]. Thus, the quantification of fat was done following the recommendations of the QIBA [18]. However, the sequences used had different echo times, which can potentially lead to a misestimation of R2* and the spectral fat components. Therefore, fat fraction could be mistakenly determined to be incorrect. We estimate this error to be minor and negligible in the applied cohort.

Finally, we used a threshold value of 5.1% to differentiate between fatty liver disease and healthy liver. Although there are slightly different cut-off values in the literature, we chose this value based on our own work comparing PDFF and histopathological fat grading using the same hardware and software [19].

## Conclusion

In conclusion, our study found that a quarter of the population developed fatty liver disease over a five-year follow-up period, which was associated with age, diabetes mellitus, low HDL-cholesterol, and diastolic blood pressure. Increased physical activity was found to have a protective effect on the development of fatty liver. Using our prediction model and formula, we can provide an individualized risk assessment for the development of NAFLD.

### Electronic supplementary material

Below is the link to the electronic supplementary material.

Supplementary Material 1

## Data Availability

The datasets used and/or analyzed during the current study are available from the corresponding author upon reasonable request (https://www.fvcm.med.uni-greifswald.de).

## Abbreviations

ALT: Alanine aminotransferase
AST: Aspartate aminotransferase
AUC: Area under the curve
BMI: Body mass index
GGT: Gamma-glutamyltransferase
HDL-C: High-density lipoprotein-cholesterol
LDL-C: Low-density lipoprotein-cholesterol
MRI: Magnetic resonance imaging
NAFLD: Non-alcoholic fatty liver disease
NASH: Non-alcoholic steatohepatitis
OR: Odds ratio
PDFF: Proton-density fat fraction
QIBA: Quantitative Imaging Biomarkers Alliance
ROC: Receiver operating characteristic
ROI: Region of interest
SHIP: Study of Health in Pomerania
T2DM: Type 2 diabetes mellitus
CI: 95% Confidence Interval
