# Supplementary material for: Exploring factors associated with non-alcoholic fatty liver disease using longitudinal MRI
Source: BMC Gastroenterol. 2024 Jul 23;24:229. doi: 10.1186/s12876-024-03300-0 (PMC11267668; doi:10.1186/s12876-024-03300-0)
Supplement: Supplementary file 1 — Supplementary Material 1 [file 12876_2024_3300_MOESM1_ESM.docx]

**Appendix / Supplemented section**

The data collected in this study have been published several times. The data collection was carried out as described in the following papers: "Hepatic steatosis and hepatic iron overload modify the association of iron markers with glucose metabolism disorders and metabolic syndrome" [1] and "Variability of biomarkers used for the classification of metabolic syndrome: A repeated measurements study" [2].

**Interview and physical examination**

All participants underwent a standardized computer-assisted personal interview, during which they provided information on sociodemographic and lifestyle factors as well as medical histories and medication use [1].

**Smoking status former, current**

These data were differentiated into current smoking as well as past smoking [1].

**Alcohol consumption**

Alcohol consumption was categorized into no (0 g/day), moderate (men 0.1 - 39.9 g/day and women 0.1 - 19.9 g/day), and high alcohol (men ≥ 40 g/ day and women ≥ 20 g/day) consumption [1].

**Physical activity**

Participants who exercised for less than an hour/week in their leisure time during summer or winter were classified as physically inactive [1].

**Diabetes known yes, Arterial hypertension**

An existing diabetes mellitus is composed of a diagnosis by a physician or an already established treatment with insulin, tablets or diet. Participants were classified as hypertensive based on blood pressure readings ≥ 140/90 mmHg or use of self-reported antihypertensive medication [1].

**Systolic blood pressure, Diastolic blood pressure**

Systolic and diastolic blood pressures were measured three times after an initial five-minute rest period on the right arm of seated individuals using a digital blood pressure monitor. Measurements were repeated by three-minute intervals. The mean of the second and third measurements was calculated and used for the present analyses [2]. If the second or third measurement contained a missing value, the value of the existing measurement was used.

Arterial hypertension was defined as elevated systolic and/or diastolic blood pressure ≥ 140/90 mmHg or intake of antihypertensive medication (ATC codes C02, C03, C04, C05, C07, C08, C09).

**BMI, Waist circumference**

Height and weight were measured for the calculation of the body mass index: BMI = weight (kg) / height^2^ (m^2^). Waist circumference was measured to the nearest 0.1 cm using an inelastic tape midway between the lower rib margin and the iliac crest in the horizontal plane with the subject standing comfortably with weight distributed evenly on both feet [2].

**Food frequency score**

The food frequency score was recorded according to Winkler et al. 1995; Lüdemann et al. 2002. To determine a score for food consumption, each category of the dietary variables (meat, sausage, fish, potatoes, dough, rice, salad, vegetables, fruits, chocolate, cake, chips, crackers, oats, eggs) was assigned an auxiliary variable according to consumption behavior and a score was calculated. The higher the score, the more recommendable the food consumption was for health [3, 4].

**Laboratory measurements**

Fasting blood samples were collected without stasis from the cubital vein following a standardized protocol, refrigerated to 4-8 °C and shipped on refrigerant packing within 4 to a maximum of 6h to the central laboratory of the University Medicine Greifswald [1]. Blood samples were taken between 7 a.m. and 11 a.m. in Vacutainer® serum gel tubes (BD, Franklin Lakes, NJ, USA) not assuring the fasting status of samples [2]. Serum levels of total cholesterol, low-density lipoprotein cholesterol (LDL-C), high-density lipoprotein cholesterol (HDL-C) and triglycerides were measured using the Dimension Vista 500 analytical system (Siemens Healthcare Diagnostics, Eschborn, Germany) [2]. Serum levels of alanine aminotransferase (ALT), aspartate aminotransferase (AST) and gamma-glutamyltransferase (GGT) were measured photometrically (Hitachi 704 and 171, Roche Diagnostics, Mannheim, Germany) [5].

Measurements of plasma fasting glucose were measured using a hexokinase method (Dimension Vista 1500, Siemens Healthcare Diagnostics, Eschborn, Germany) [1].

**Supplementary results**

The goal of our study was to identify participants with a relevant increase in PDFF. Therefore, we decided on the following definition for the follow-up of a volunteer who developed fatty liver disease: PDFF > 5.1% and an increase of > 37% over the study period. As recommended by the Quantitative Imaging Biomarkers Alliance (QIBA) a PDFF change of ± 5% (absolute difference) can be considered relevant [6]. As stated in the methodology section, an additional calculation, defined as individuals with a PDFF > 5.1% and an increase of 5% in liver fat content from baseline, was carried out.

**Table A.1: Characteristics of participants without fatty liver disease collected at baseline and corresponding associations for participants who did not develop fatty liver (group A) and developed fatty liver in follow-up (group B). Each variable was tested independently.**

OR = odds Ratio, p = level of significance

|  | **Cohort A:**  No fatty liver in follow-up,  n = 136 | **Cohort B:**  Relevant fatty liver in follow-up,  n = 54 |  | **OR** | **p** | **95 % Confidence interval** |
| --- | --- | --- | --- | --- | --- | --- |
| **Age (years)** | **51.93 ± 12.20** | **58.65 ± 11.87** |  | **1.04** | **0.002** | **1.01-1.08** |
| **BMI (kg/m^2)** | **25.82 ± 3.65** | **27.44 ± 3.41** |  | **1.11** | **0.046** | **1.01-1.23** |
| **Waist circumference (cm)** | **83.35 ± 10.50** | **90.61 ± 8.94** |  | **1.05** | **0.024** | **1.01-1.09** |
| **Food frequency score** | 14.28 ± 3.17 | 14.48 ± 3.29 |  | 1.03 | 0.602 | 0.91-1.17 |
| **Alcohol consumption (g/day)** | 8.87 ± 10.34 | 8.67 ± 11.55 |  | 0.97 | 0.108 | 0.93-1.01 |
| **Glucose i.S. (mmol/l)** | 5.33 ± 0.68 | 5.42 ± 0.81 |  | 0.91 | 0.714 | 0.55-1.50 |
| **Cholesterol i.S. (mmol/l)** | 5.43 ± 1.13 | 5.58 ± 0.99 |  | 1.05 | 0.765 | 0.76-1.46 |
| **LDL-Cholesterol i.S. (mmol/l)** | 3.28 ± 0.83 | 3.49 ± 0.82 |  | 1.21 | 0.389 | 0.79-1.86 |
| **HDL-Cholesterol i.S. (mmol/l)** | 1.60 ± 0.38 | 1.45 ± 0.30 |  | **0.28** | **0.032** | **0.09-0.90** |
| **Triglycerides (mmol/l)** | 1.35 ± 0.75 | 1.73 ± 0.91 |  | 1.23 | 0.371 | 0.78-1.93 |
| **ALT i.S. (µmol/sl)** | 0.36 ± 0.19 | 0.41 ± 0.16 |  | 2.39 | 0.361 | 0.37-15.61 |
| **AST i.S. (µmol/sl)** | 0.29 ± 0.11 | 0.31 ± 0.12 |  | 0.76 | 0.872 | 0.03-20.52 |
| **GGT (µmol/sl)** | 0.54 ± 0.43 | 0.66 ± 0.70 |  | 1.19 | 0.588 | 0.64-2.21 |
| **Systolic blood pressure (mm/Hg)** | 126 ± 17 | 134 ± 15 |  | 1.01 | 0.305 | 0.99-1.04 |
| **Diastolic blood pressure (mm/Hg)** | 77 ± 9 | 82 ± 9 |  | **1.04** | **0.049** | **1.00-1.09** |
| **Smoking status**  **former**  **current** | 51 (35.7%)  32 (22.4%) | 20 (43.5%)  8 (17.4%) |  | 0.78  1.01 | 0.562  0.990 | 0.33-1.82  0.36-2.82 |
| **Arterial hypertension** | 46 (33.8%) | 34 (63.0%) |  | 2.03 | 0.070 | 0.94-4.36 |
| **Male gender** | 47 (34.6%) | 30 (55.6%) |  | 1.96 | 0.056 | 0.98-3.93 |
| **Diabetes known yes** | 1 (0.7%) | 4 (7.4%) |  | 8.12 | 0.091 | 0.71-92.38 |
| **Physical activity yes** | **108 (80.0%)** | **39 (72.2%)** |  | 0.54 | 0.167 | 0.23-1.29 |

Data are expressed as mean ± standard deviation (continuous variables) or as absolute numbers and percentages (categorical variables). Odds ratios (OR) are derived from logistic regression models adjusted for age, sex, and liver fat content at baseline.

**Table A.2: Variables kept in the final prediction model for incident fatty liver.**

OR = odds Ratio, p = level of significance

|  | **Odds Ratio** | **p** | **95 % Confidence interval** |
| --- | --- | --- | --- |
| **Age** | **1.07** | **< 0.001** | **1.03-1.10** |
| **Physical activity yes** | **0.41** | **0.039** | **0.17-0.96** |
| **Diastolic blood pressure** | **1.06** | **0.002** | **1.02-1.11** |
| **HDL-cholesterol i.S.** | **0.19** | **0.003** | **0.06-0.57** |

Results are derived from a logistic regression model.

The formula for calculating the individual risk for incident fatty liver is:

*“1/(1+1/exp(-6.31337 + 0.0656828*age (years) – 0.8948157*(if physically active) + 0.0622572*diastolic blood pressure (mmHg) – 1.661238*HDL-cholesterol))”.*

The discrimination of the model was evaluated using ROC analysis, and the AUC was found to be 0.7597 (95% Confidence interval: 0.6899; 0.8294).

**Discussion of the supplementary results**

The results of these additional analyses demonstrate comparable findings to the primary analyses. However, in the prediction model, the factor 'type 2 diabetes' is not retained and exhibits no significant influence on the prediction.

**References**

1. Pitchika A, Kühn J-P, Schipf S, et al (2021) Hepatic steatosis and hepatic iron overload modify the association of iron markers with glucose metabolism disorders and metabolic syndrome. Liver Int 41:1841–1852

2. Ittermann T, Dörr M, Markus MRP, Nauck M, Jürgens C, Schipf S, Schmidt CO, Völzke H, Richter A (2022) Variability of biomarkers used for the classification of metabolic syndrome: A repeated measurements study. Nutr Metab Cardiovasc Dis 32:1693–1702

3. Winkler G, Döring A, Keil U (1995) [Mealtime patterns in a southern German population. Results from the WHO MONICA 1984/1985 Augsburg nutritional survey project]. Z Ernahrungswiss 34:2–9

4. Luedemann J, Schminke U, Berger K, Piek M, Willich SN, Döring A, John U, Kessler C (2002) Association between behavior-dependent cardiovascular risk factors and asymptomatic carotid atherosclerosis in a general population. Stroke 33:2929–2935

5. Baumeister SE, Völzke H, Marschall P, John U, Schmidt C, Flessa S, Alte D (2008) Impact of Fatty Liver Disease on Health Care Utilization and Costs in a General Population: A 5-Year Observation. Gastroenterology 134:85–94

6. QIBA Proton Density Fat Fraction Biomarker Committee (2023) MRI-Based Proton Density Fat Fraction (PDFF) of the Liver. https://qibawiki.rsna.org/index.php/Profiles. Accessed 27 May 2024
